# OA01 Tennis shoulder? A rare case of brachial neuritis

**DOI:** 10.1093/rap/rkad070.001

**Published:** 2023-09-27

**Authors:** Sophia Kostich, Rizwan Rajak

**Affiliations:** Croydon University Hospital, London, United Kingdom; Croydon University Hospital, London, United Kingdom

## Abstract

**Introduction:**

Sports and exercise medicine (SEM) is an ever-expanding medical discipline often requiring input from various specialities. Sports injuries can be hard to diagnose and may mimic autoimmune conditions. Consequently, rheumatologists and their knowledge of diagnostics and management can aid significantly.

Here we present a case of an acute upper limb sporting pathology. Whilst the patient presented with symptoms consistent with a regional MSK injury, further investigation demonstrated brachial neuritis (BN). This case is of interest as the differentials were broad and it was an extremely challenging condition to diagnose. The final diagnosis was a rare disorder of poorly understood aetiology.

**Case description:**

A 73-year-old gentleman presented to clinic with a 9-month history of right scapular pain. He complained of sharp pain in the right scapular region, as well as needing to repeatedly reposition from right to left at night. The sharp pain was intermittent and brief, but he had a more persistent dull ache around the medial and inferior aspect of the scapular. He had used various forms of pain relief including ibuprofen, co-codamol/paracetamol and acupuncture, chiropractors and osteopathy - all of which had only provided short term relief. There had been no trauma to the shoulder. Examination showed mild right scapular winging, rhomboid muscle spasm, reduced range of movement in cervical spine lateral flexion. Of note he was normally fit and active, exercising and playing tennis regularly. He also suffered from hypertension and ischaemic heart disease.

Investigations were performed including an EMG, MRI of his chest wall and pain x-ray of his thoracic spine. X-ray of the spine showed DISH, with right sided osteophytes over consecutive vertebrae and signs of disc degeneration. MRI of his spine confirmed the disc changes as well. Of note, MRI of his spine showed posterior para-spinal muscle asymmetry concentrated in the trapezius muscle, but there was no evidence of muscle oedema or fatty changes. EMG was performed and suggested a likely diagnosis of BN. As such the overall impression was tennis training induced BN causing paraspinal muscle spasm.

The patient was initially advised to take pregabalin 50mg twice daily for one month and diazepam 2mg twice daily for one month. A tapering dose was recommended, however the patient highlighted he had not used any medications to relieve his pain and did not require repeat prescription. He was referred for physiotherapy which provided significant resolution to his symptoms.

**Discussion:**

This was an interesting and challenging diagnostic case. There were various important clinical decisions which aided diagnosis. Patients with BN will often experience debilitating and painful symptoms. BN symptoms can often go through phases including acute pain, a period of weakness due to muscle atrophy and a recovery phase with improvement of weakness. Simple investigations such as MRI will not aid in diagnosis. It is important to consider further investigations where diagnoses are unclear, with neurophysiological studies being an often underutilised but high yield option. Despite an unclear diagnosis, early referral to physiotherapy in this case enabled rapid resolution of pain and symptoms. As the recovery process often occurs over months to years, longer-term treatments are an important topic for further discussion.

There are some other interesting aspects and learning points. Firstly, BN is a rare and poorly understood condition. Aetiology is often unknown, although in this case can likely be attributed to exercise. The history the patient gave was in keeping with symptoms intensifying in line with his exercise intensity increasing. Existing case reports of sports-induced BN primarily focus on athletes, and it is a less commonly documented cause of BN. Whilst perhaps less so in this case, it is important to consider the possibility that BN, even in the context of being related to sporting activities, is more commonly idiopathic or infection-related.

Secondly, initial differentials were extremely broad with other differentials being much more likely. From initial assessment, the general impression was mechanical rhomboiditis with scapular winging secondary to overuse from tennis. Prevalence of mechanical musculoskeletal pain is up to 49% in the general population, whilst BN is estimated to have a population prevalence of 1.64 per 100,000. Where symptoms are not explained by initial history and imaging, it is important to consider rarer differentials such as BN.

**Key learning points:**

There are two key learning points that can be taken from this case:

Firstly, it is important to acknowledge how broad the differentials are for regional SEM injuries. Whilst BN can be related to stressful exercise, it is more commonly secondary to infection, autoimmune conditions, or idiopathic. This made it a much less likely differential compared to more common sport related injuries. Additionally, it highlights the importance of further investigation where the diagnosis is unclear and the relevance of neurophysiological studies in SEM. A topic of further discussion includes key differentials for this presentation, both more common and less common, as a tool for others to utilise when seeing similar cases in the future.

Secondly, whilst this case is an acute presentation, brachial neuritis is often chronic and can take years to recover from. Some key learning points relate to the management of this condition in both phases. Whilst neuropathic agents are often used, as the acute phase is often limited to a few weeks, faster acting analgesics have been shown to be more effective due to the slow onset of neuropathic agents. However, during the subsequent weeks and due to the chronicity of the conditions, physiotherapy has been shown to play a key role in improvement of symptoms. This case provides interesting insight into the importance of both appropriate analgesia combined with physiotherapy input. It also suggests that further treatments may be appropriate for more chronic cases of brachial neuritis, and indeed longer-term treatments for many SEM presentations, including transcutaneous electrical nerve stimulation, anti-inflammatory treatment such as steroids or IVIG, and even surgical interventions.

